# A procedure and model for the identification of uni- and biarticular structures passive contribution to inter-segmental dynamics

**DOI:** 10.1038/s41598-023-37357-w

**Published:** 2023-06-29

**Authors:** Axel Koussou, Raphaël Dumas, Eric Desailly

**Affiliations:** 1 Fondation Ellen Poidatz, Pôle Recherche & Innovation, 77310 Saint-Fargeau-Ponthierry, France; 2 Univ Lyon, Univ Gustave Eiffel, Univ Claude Bernard Lyon 1, LBMC UMR T9406, 69622 Lyon, France

**Keywords:** Musculoskeletal system, Neurological disorders, Paediatrics, Therapeutics, Orthopaedics, Biomarkers, Paediatric research, Biomedical engineering, Mechanical engineering

## Abstract

Inter-segmental moments come from muscles contractions, but also from passive moments, resulting from the resistance of the periarticular structures. To quantify the passive contribution of uni- and biarticular structures during gait, we propose an innovative procedure and model. 12 typically developed (TD) children and 17 with cerebral palsy (CP) participated in a passive testing protocol. The relaxed lower limb joints were manipulated through full ranges of motion while kinematics and applied forces were simultaneously measured. The relationships between uni-/biarticular passive moments/forces and joint angles/musculo-tendon lengths were modelled by a set of exponential functions. Then, subject specific gait joint angles/musculo-tendon lengths were input into the determined passive models to estimate joint moments and power attributable to passive structures. We found that passive mechanisms contribute substantially in both populations, mainly during push-off and swing phases for hip and knee and push-off for the ankle, with a distinction between uni- and biarticular structures. CP children showed comparable passive mechanisms but larger variability than the TD ones and higher contributions. The proposed procedure and model enable a comprehensive assessment of the passive mechanisms for a subject-specific treatment of the stiffness implying gait disorders by targeting when and how passive forces are impacting gait.

## Introduction

Human joints are complex mechanisms made of a set of structures allowing their proper functioning and preservation. Some are passive, such as the skin, ligaments, tendons, joint capsule. Others can be active, namely the muscle tissue under contraction. Inter-segmental moments computed by inverse dynamics are the result of both deformations of the periarticular structures and contractions of muscles. In the absence of muscular contraction, tissue deformations generate resistance to movement and produce the so-called passive moments.

These passive moments have been measured in-vivo in several studies using isokinetic dynamometer or custom made device by measuring the necessary passive forces to mobilize the joint^1–5^. Those studies have highlighted the increase of the passive moments as a function of the joint angle, up to significant values at the end of the range of motion. Then, some authors have identified mathematical models, usually exponential, of the passive moment-joint angle relationship^1,4–6^. These models suggest that substantial passive moments could be present in normal gait, and may be influenced by the stretch of biarticular muscles^7–9^.

Moreover, passive joint resistance is often cited as a contributing factor to various gait impairments. Limited passive range of motion (ROM) can partly explain the gait deviations in children with cerebral palsy (CP). For example, hip flexor passive stiffness may limit step length or excessive ankle plantarflexor passive stiffness can limit dorsiflexion and thereby contributes to equinus gait patterns^10,11^. Nevertheless, to our knowledge, Tardieu et al.^12^ are the only authors who evaluated the passive moments during gait for this population and for the ankle only. This lack of objective assessments may be caused by the technical difficulties and the time needed to achieve such measurements. For example, Whittington et al.^9^ have proposed a model which quantitatively estimate the contribution of uni- and biarticular passive mechanisms to lower limbs joints kinetics. Nevertheless, the identification of representative model parameters, distinguishing uni- and biarticular structures, require fifteen different joint mobilizations, which represent an important amount of time, making its clinical implementation difficult. Choosing a limited number of positions where resistance of biarticular muscles can be extracted and using a musculoskeletal model to extract the muscle–tendon unit lengths, in order to obtain, not the passive moment-joint angle relationship, but the passive force-muscle tendon unit length relationship, can be a solution to reduce the number of positions to be tested.

The objective of this study was to propose and evaluate a procedure limiting the number of positions tested and a model to obtain the contribution to hip, knee and ankle inter-segmental moments and powers of uni- and biarticular passive structures in typically developing (TD) and CP children.

## Method

### Subjects

12 TD children (mean age: 11.1 ± 2.80; 6 males) and 17 children with CP (mean age: 13.6 ± 2.17; 10 males; GMFCS I:5 II:11 III:1) participated to the study. The protocol was approved by the ethical committee of the Comité de Protection des Personnes—Ouest IV and voluntary adhesion of all the participants and informed consent of the legal guardians were attested. All methods were performed in accordance with the relevant guidelines and regulations. This study was registered in the ClinicalTrials.gov database (NCT04596852).

### Experimental procedure

The experimental procedure included two steps: a passive testing protocol and a gait analysis. Throughout the procedure, 3D body segment kinematics (100 Hz) were determined using a marker set placed over specific body landmarks (Plug-in-Gait marker set with two additional markers placed over the iliac bone to take into account that the posterior superior iliac markers are not visible when the patient is lying) and motion analysis system (15 cameras VICON, Oxford, UK). EMG activities were synchronously recorded using pre-amplified dual differential surface electrodes (DE-2.1, DelSys, Inc., Boston, MA, 2000 Hz) placed, bilaterally, over the rectus femoris, vastus lateralis, semitendinosus, tibialis anterior, peroneus longus, soleus and gastrocnemius lateralis muscles. Electrode locations were determined according to the Surface Electromyography for the Non-Invasive Assessment of Muscles (SENIAM) guidelines and prepared by shaving the skin and cleaning with alcohol.

Firstly, each subject performed a series of gait trials at their self-selected speed along a 10 m walkway. Ground reaction forces (2000 Hz) were recorded using four imbedded forceplates (2 AMTI, Waterfown, USA and 2 Kistler, Hampshire, UK).

**Figure 1:**
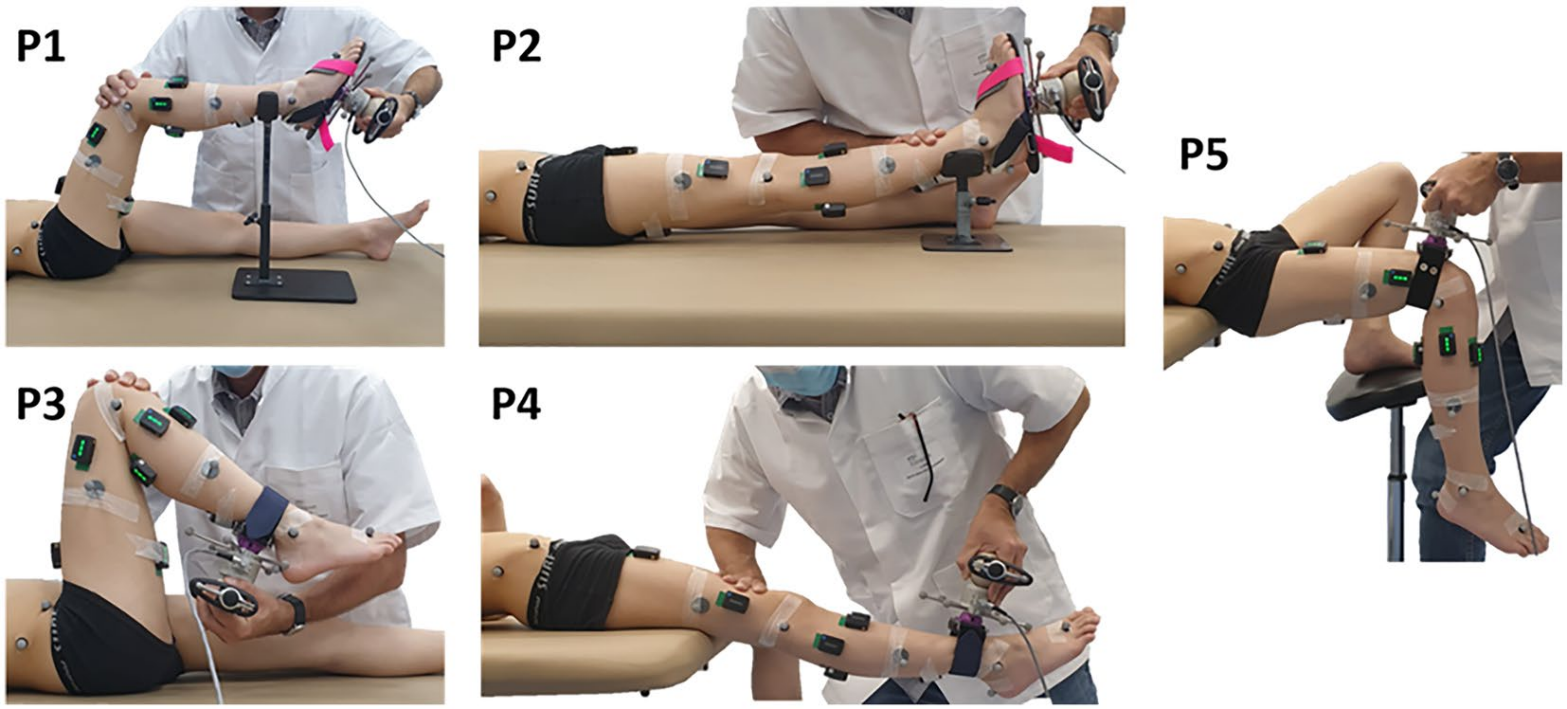
Test positions. Joints were mobilized through sagittal range of motion using a 3D handheld dynamometer. Dynamometer and body segment kinematics were measured with reflective markers and motion analysis system. Muscle activity was controlled with surface electromyography.

Secondly, a passive testing protocol was designed to obtain continuous joint angle and joint resistance measurements. Participants were asked to relax, while the assessor performed subject’s joint mobilizations at a low velocity (inferior to 10°/s) through full available sagittal range of motion (ROM), using a 3D handheld dynamometer (Sensix, FR, 2000 Hz) (Fig. 1). Six markers, rigidly attached to the dynamometer, were used to continuously track the position and orientation of the device relative to the subject’s segments. The joints were mobilized 3 times in five different supine positions to ensure the characterization of the entire lower limb joints taking into account the stretch of the biarticular muscles:

- Ankle with the knee at 90° and 0° (P1, P2).
- Knee with the hip at 90° and 0° (P3, P4).
- Hip with free knee (P5).

EMG signals were visually monitored during passive testing and any trials with detectable muscle activity (appearance of clear bursts) was redone.

### Biomechanical model and analysis

A seven segment, 24 degree-of-freedom model of the pelvis and lower extremities was used to characterize joint kinematics. Six degrees of freedom were used to define the position and orientation of the pelvis. Three degrees of freedom were used to define the orientation of each lower limb joints. Body segment coordinate systems, joint centers and segment lengths were established using the marker positions collected during a calibration trial. Then, joint angles during gait or passive trials were calculated using the Cardan angles^13^.

Inverse dynamics analysis, using homogeneous matrix, was used to compute the hip, knee, and ankle inter-segmental moments from the joint kinematics and measured forces from the handheld dynamometer (passive testing) or ground forceplates (gait testing)^14,15^.

Moreover, muscle–tendon unit (MTU) length and moment arms during passive or gait testings, were computed via OpenSim with an adult generic model^16^, by using the measured kinematics and after having scaled the model, taking into account the tibial and femoral torsions determined during clinical examination^17^. To generate accurate muscle–tendon length estimates, we modified the OpenSim model with segment coordinate systems and joint degrees of freedom similar to our biomechanical model. Degrees-of-freedom have been added to the ankle and the knee joints to model spherical joints driven by the experimental 3D joint angles, and the pelvis joint was tilted^18^.

#### Passive moment model

The relationships between passive moments and sagittal joint angles and between passive forces and MTU lengths were modeled by exponential functions^6,19,20^ (Supplementary material—S1).

The model was developed to account for the stretch of uni- and biarticular muscles about the hip, knee, and ankle. The model included 3 biarticular muscles assimilated to their biarticular agonists groups: gastrocnemius lateralis muscle (*Gas*) for both gastrocnemii, semitendinosus (*Se*) for hamstrings, and rectus femoris (*Rf*).

##### Ankle

At P1, we assumed that only single-joint structures of the ankle produce resistance. Thus, from the computed passive moment, we modelled the uniarticular moment of the ankle, ${MA}_{Uni}$, relatively to the ankle angle, through a double exponential. This passive moment is due to the ankle uniarticular dorsi- and planterflexors.

At P2, we assumed that the biarticular structures of the ankle (*Gas*) also produce resistance. Thus, from computed passive moment, ${M}_{P2}$, and by subtracting the uniarticular moment of the ankle, ${MA}_{Uni}$, we determined ${MA}_{Gas}$, the biarticular moment. Then, from the determined *Gas* moment arm over the ankle flexion/extension, ${m}_{Gas/a}$, during P2, we determined ${F}_{Gas}$.

$${F}_{Gas}=\frac{{M}_{Gas}}{{m}_{Gas/a}}=\frac{{M}_{P2}-{MA}_{Uni}({\theta }_{A})}{{m}_{Gas/a}}$$

Similarly, knowing the *Gas* MTU length during P2, we were able to model this length–force relationship as a simple exponential.

##### Knee

First, we subtracted to the computed knee flexion moment at P3 and P4, the moment coming from the *Gas* thanks to the modelled length–force relationship and to the *Gas* MTU length, and moment arm over the knee flexion/extension, ${m}_{Gas/k}$. The relationships between these adjusted moments (denoted P3′ and P4′) and joint angles were modelled with double exponentials.

Then, we determined the uniarticular moment of the knee, ${MK}_{Uni}$, from these adjusted functions without *Gas* resistance. We assumed that ${MK}_{Uni}$ was null over most of the ROM, but equal to P3′ when an extension moment was present and equal to P4′ when a flexion moment was present. These two conditions were encountered at the limits of the ROM, where the biarticular structures are flexed enough not to produce any resistance.

Finally, as previously, we determined knee biarticular resistance for *Se* and *Rf* muscles, ${F}_{Se}$, ${F}_{Rf}$.

$$F_{Se} = \frac{{M_{Se} }}{{m_{Se/k} }} = \frac{{M_{{P3^{\prime}}}- MK_{Uni} (\theta_{K} )}}{{m_{Se/k} }}$$

$$F_{Rf} = \frac{{M_{Rf} }}{{m_{Rf/k} }} = \frac{{M_{P4^{\prime}} -MK_{Uni} (\theta_{K} )}}{{m_{Rf/k} }}$$

where ${m}_{Se/k}$, ${m}_{Rf/k}$ are the knee flexion/extension moment arms of the *Se* and *Rf*.

Knowing their MTU lengths during P3 and P4, we modelled these length-tension relationships with simple exponential.

##### Hip

From the computed hip flexion moment at P5,${M}_{P5}$, we determined the uniarticular moment of the hip, ${MH}_{Uni}$. We subtracted to ${M}_{P5}$ the hip moment due to the *Se* and *Rf* that we determined thanks to the modelled length–force relationships and to their MTU lengths, and moment arm over the hip flexion/extension, ${m}_{Se/h}$ and ${m}_{Rf/h}$.

$${MH}_{Uni}\left({\theta }_{H}\right)={M}_{{P}_{5}}-{F}_{Se}\left({L}_{Se}\left({\theta }_{H},{\theta }_{K}\right)\right)*{m}_{Se/h}-{F}_{Rf}\left({L}_{Rf}\left({\theta }_{H},{\theta }_{K}\right)\right)* {m}_{Rf/h}$$

Finally, ${MH}_{Uni}\left({\theta }_{H}\right)$ was modelled with a double exponentials.

Model parameters were estimated for each subject using a least square fitting method that minimized the sum of squared differences between the computed (inverse dynamics) and predicted (exponential models) passive moments (fmincon, MATLAB, The Mathworks Inc.).

#### Contribution during gait

Passive moments during gait, for the different lower limb joints, were predicted using the joint angles, MTU lengths and moment arms measured during gait as inputs to the previous identified models:

$${MH}_{Gait}={MH}_{Uni}\left({\theta }_{{H}_{Gait}}\right)+{F}_{Se}\left({L}_{Se}\left({\theta }_{{H}_{Gait}},{\theta }_{{K}_{Gait}}\right)\right)*{m}_{Se/h}\left({\theta }_{{H}_{Gait}},{\theta }_{{K}_{Gait}}\right)+{F}_{Rf}\left({L}_{Rf}\left({\theta }_{{H}_{Gait}},{\theta }_{{K}_{Gait}}\right)\right){* m}_{Rf/h}\left({\theta }_{{H}_{Gait}},{\theta }_{{K}_{Gait}}\right)$$

$${MK}_{Gait}={MK}_{Uni}\left({\theta }_{{K}_{Gait}}\right)+{F}_{Se}\left({L}_{Se}\left({\theta }_{{H}_{Gait}},{\theta }_{{K}_{Gait}}\right)\right)*{m}_{Se/k}\left({\theta }_{{H}_{Gait}},{\theta }_{{K}_{Gait}}\right)+{F}_{Rf}\left({L}_{Rf}\left({\theta }_{{H}_{Gait}},{\theta }_{{K}_{Gait}}\right)\right)*{m}_{Rf/k}\left({\theta }_{{H}_{Gait}},{\theta }_{{K}_{Gait}}\right) +{F}_{Gas}\left({L}_{Gas}\left({\theta }_{{K}_{Gait}},{\theta }_{{A}_{Gait}}\right)\right)*{m}_{Gas/k}\left({\theta }_{{K}_{Gait}},{\theta }_{{A}_{Gait}}\right)$$

$${MA}_{Gait}={MA}_{Uni}\left({\theta }_{{A}_{Gait}}\right)+{F}_{Gas}\left({L}_{Gas}\left({\theta }_{{K}_{Gait}},{\theta }_{{A}_{Gait}}\right)\right)*{m}_{Gas/a}\left({\theta }_{{K}_{Gait}},{\theta }_{{A}_{Gait}}\right)$$

These predicted passive moments, as well as inter-segmental moments computed during gait, were multiplied by their respective joint angular velocities to determine passive and inter-segmental powers. To understand the relative timing and role of the individual passive structures included in the passive model, we also separately computed the passive power due to uni- and biarticular structures. The convention used to represent the curves of moments corresponds to that recommended by the ISB^21^. The reader can invert the ankle and hip curves to find a convention more generally used by the clinicians. In order to be able to compare our results with the literature and to compare CP with TD, passive contribution to inter-segmental moments and powers are compared at different instants of the gait cycle over the mean curves of both populations.

## Results

For CP children, 2, 1 and 4 trials have been excluded due to some experimental issues (multiple occlusion of markers or muscle activity) for P2, P3 and P5 respectively. Therefore passive contributions to inter-segmental moments and powers were calculated at the ankle, knee and hip for 15, 16 and 13 subjects respectively. For TD children, no experimental issues were encountered and passive contributions to inter-segmental moments and powers were calculated for all the 12 subjects.

The exponential models identified for each subject were able to reproduce accurately the passive joint resistance from the measured joint angles or MTU lengths (Supplementary material—S2 and Supplementary Material 2).

### TD children

**Figure 2:**
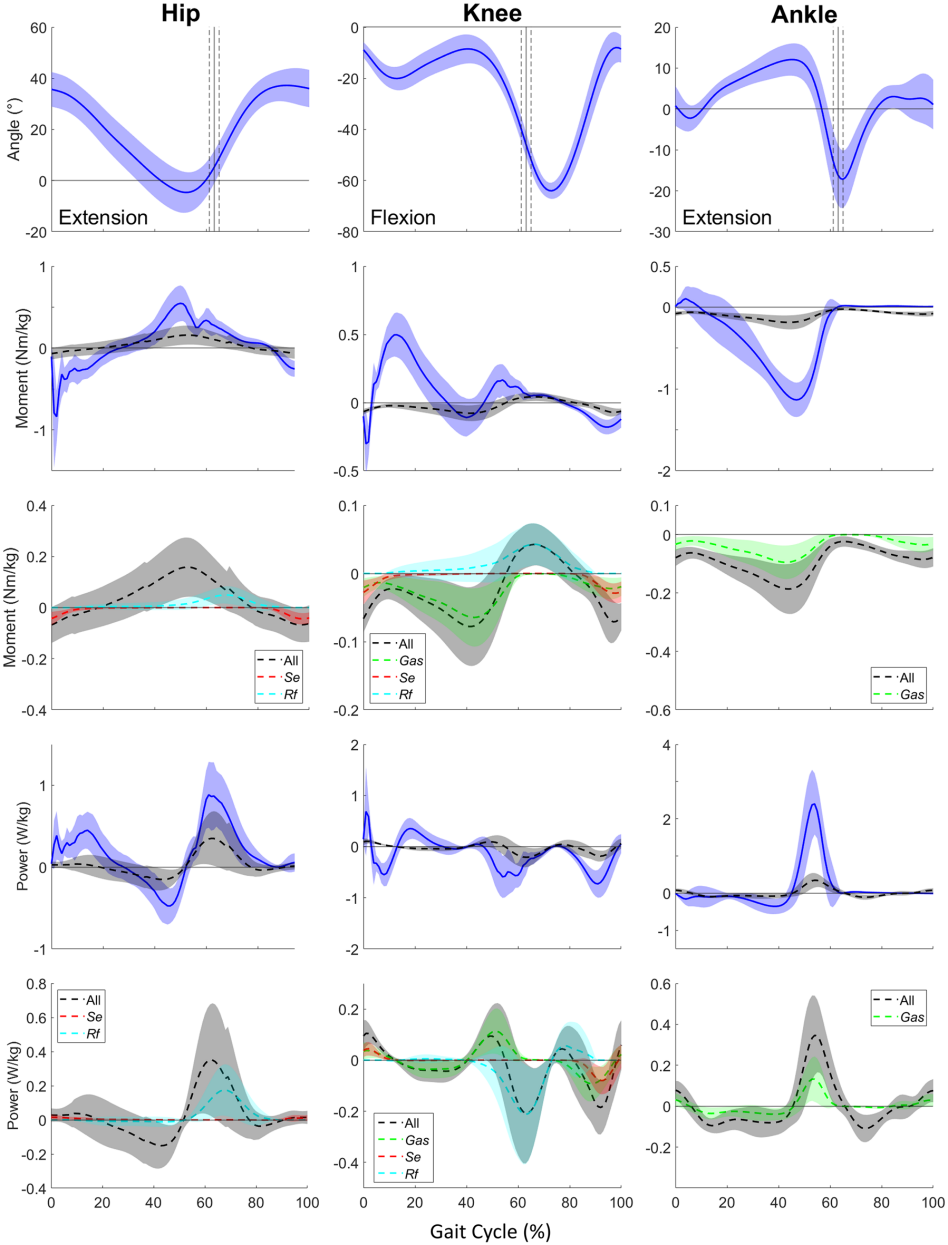
Ensemble-averaged joint angle, inter-segmental and passive (detailed between uni- and biarticular muscles) moment, and inter-segmental and passive (detailed between uni- and biarticular muscles) power curves from TD children during gait. Toe-off timing (± 1 SD) is indicated with a straight vertical line.

Joint angles, inter-segmental moments and powers, with details of passive structures, during gait are presented in Fig. 2 for TD children. Passive moments contribute substantially at different instants of the gait cycle for the lower limb joints.

#### Hip

At the hip, the main passive moment contributions to intersegmental ones were from mid-stance through initial swing phase and during late swing, respectively for ~ 27% and ~ 26% of the peak values (Fig. 2(2,1)). The first passive contribution mainly comes from uniarticular structures of the hip but at the end of the phase *Rf* also contribute for ~ 69% of the passive moment, whereas the latter is mainly due to *Se* (Fig. 2(3,1)). In terms of power, passive contributions were found during two power bursts at single support and early swing phases, representing respectively ~ 30% and ~ 39% of the peak values (Fig. 2(4,1)). It appears that, the first power contribution comes from uniarticular structures, whereas the latter is mainly due to uniarticular structures and then to *Rf* (Fig. 2(5,1)).

#### Knee

At the knee, the main passive moment contributions to intersegmental ones were during single support, early swing and late swing phases, respectively for ~ 95% , ~ 99% and ~ 40% (Fig. 2(2,2)). These passive contributions are mainly due to *Gas*, to *Rf* and to both *Gas* and *Se,* respectively (Fig. 2(3,2)). In terms of power, passive contributions were found during three power bursts during loading response, push-off and late swing phases, representing respectively ~ 10%, ~ 33% and ~ 25% of the peak values (Fig. 2(4,2)). It appears that, the first power contribution comes from *Gas* and *Se*, the second from the *Rf* whereas the latter is mainly due to *Gas* and *Se* (Fig. 2(5,2)). It can be noted that the passive power peak at push-off is slightly later than the inter-segmental one.

#### Ankle

At the ankle, the main passive moment contribution to intersegmental moment were during push-off phase for ~ 17% of the peak value (Fig. 2(2,3)). This passive contribution is shared between the uniarticular structures and *Gas* (Fig. 2(3,3)). Moreover, during swing phase a passive moment of ~ 0.05 Nm/kg, shared between the uniarticular structures and *Gas* is found, while the inter-segmental moment is null. In terms of power, passive contributions were found during two power bursts during mid and late stance and push-off phases, representing respectively ~ 20%, ~ 15% of the peak values (Fig. 2(4,3)). It appears that, these passive contributions are shared between the uniarticular structures and *Gas* (Fig. 2(5,3)).

### CP children

**Figure 3:**
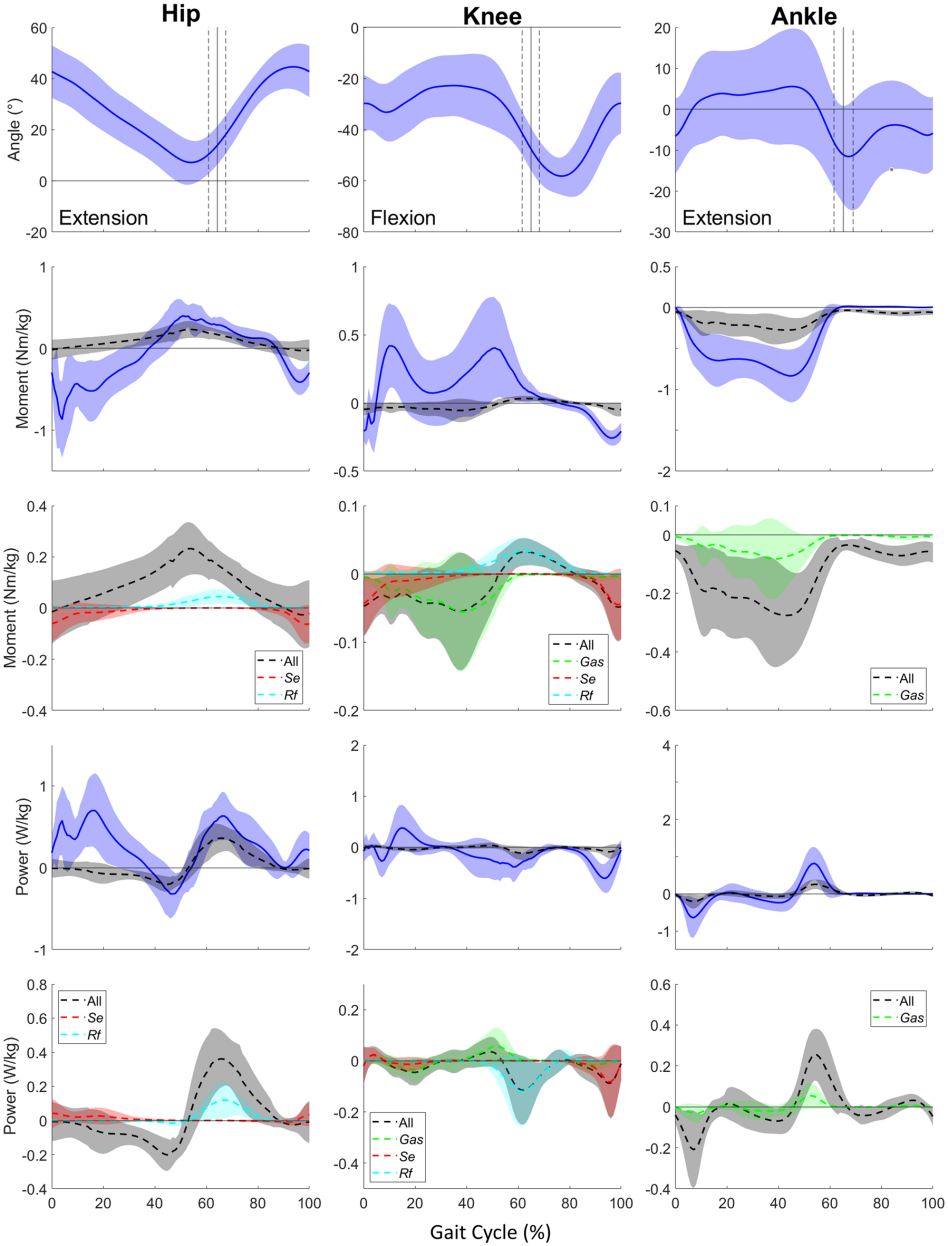
Ensemble-averaged joint angle, inter-segmental and passive (detailed between uni- and biarticular muscles) moment, and inter-segmental and passive (detailed between uni- and biarticular muscles) power curves from CP children during gait. Toe-off timing (± 1 SD) is indicated with a straight vertical line.

Joint angles, inter-segmental moments and powers, with contribution of passive structures, during gait are presented in Fig. 3 for children with CP. Among other disorders, CP children showed a lack of dorsiflexion and knee extension in mid-stance and swing phase as well as a lack of hip extension in late stance (Fig. 3). They also showed a larger inter-subject variability as depicted by the larger standard deviation bands, showing the heterogeneity of these subjects.

Although their inter-segmental moments were different from the ones of the TD children, their average passive moments and powers were comparable, and the share between uni- and bi-articular structures was similar (Figs. 2–3). However, at the ankle there is a slight difference during loading response, where passive moment is positive for TD children while it is negative for CP children. This difference is attributable to a fore-foot initial contact in the CP group. Due to altered inter-segmental moments and powers, some previously determined inter-segmental peaks were not present in CP gait. Thus, no contribution was determined for the knee moment peak during single-support phase and for the knee power peak during loading response.

#### Hip

At the hip, the main passive moment contributions to intersegmental ones were from mid-stance through initial swing phase and during late swing, respectively for ~ 57% and ~ 10% of the peak values (Fig. 3(2,1)). The first passive contribution mainly comes from uniarticular structures of the hip but at the end of the phase *Rf* also contribute for ~ 16% of the passive moment, whereas the latter is mainly due to *Se* (Fig. 3(3,1)). In terms of power, passive contributions were found during two power bursts at single support and push-off phases, representing respectively ~ 65% and ~ 57% of the peak values (Fig. 3(4,1)). It appears that, the first power contribution comes from uniarticular structures, whereas the latter is mainly due to uniarticular structures and then to *Rf* (Fig. 3(5,1)).

#### Knee

At the knee, the main passive moment contributions to intersegmental ones were during early swing and late swing phases, respectively for ~ 99% and ~ 20% (Fig. 3(2,2)). These passive contributions are mainly due to *Rf* and to *Se* respectively (Fig. 3(3,2)). In terms of power, passive contributions were found during two power bursts during push-off and late swing phases, representing respectively ~ 33% and ~ 13% of the peak values (Fig. 3(4,2)). It appears that, the first power contribution comes from the *Rf* whereas the latter is mainly due to the *Se* (Fig. 3(5,2)).

#### Ankle

At the ankle, the main passive moment contribution to inter-segmental moment were during push-off phase for ~ 32% of the peak value (Fig. 3(2,3)). This passive contribution is shared between the uniarticular structures and *Gas* (Fig. 3(3,3)). Moreover, during swing phase a passive moment of ~ 0.06 Nm/kg, mainly coming from the uniarticular structures is found, while the inter-segmental moment is null.

In terms of power, passive contributions were found during three power bursts during loading response, mid and late stance and push-off phases, representing respectively ~ 31%, ~ 29%, ~ 31% of the peak values (Fig. 3(4,3)). It appears that, these passive contributions are shared between the uniarticular structures and *Gas* (Fig. 3(5,3)). The latter are also found in TD children but not the first one.

## Discussion

This study proposed and evaluated a procedure and model to obtain the contribution of uni- and biarticular passive structures to lower limb kinetics for TD and CP children, while limiting the number of positions tested.

### Procedure and model

Our procedure requires only 5 positions to obtain the contribution of passive mechanisms to inter-segmental moments with distinction between uni- and biarticular structures. To our knowledge, Whittington et al.^9^ are the only one to also propose such a model. The procedure and model presented here are less time-consuming than the ones of Whittington et al.^9^, which require 15 positions with several joint configurations. Concerning our joint mobilization positions, although some of them do not enable to test the joint over the full ROM (P4, P5), the proposed double exponential model qualitatively fit the experimental data over the joint ROM achieved during gait (Supplementary material—S2). Moreover, the hip mobilization (P5) might not be optimal to enable the complete relaxation of the subjects with CP. Indeed, in this position for 4 subjects among 17, we were unable to measure passive moment due to activity of the thigh muscles. For TD subjects, we have never encountered such cases.

Our method uses musculoskeletal modelling to obtain muscle–tendon unit length and moment arms of biarticular muscles of interest after model-scaling. Thus, we are able to obtain one length–force relationship by muscles group instead of angle-moment relationships depending on several joint angles. This innovative procedure enable to obtain subject-specific contribution of uni- and biarticular structures. No distinction is made between muscle length and tendon length and a unique stiffness is considered for the two spring in series. Tendon compliance is therefore ignored during gait. Two reasons explain this choice. First, some authors consider that for slow activities, such as gait, use of a rigid tendon is justifiable because it produces muscle force estimates close to those produced by a compliant tendon model^22^. Second, the method relies only on MTU lengths and levers arms and do not include any personalization of the muscle–tendon properties or Hill-type parameters, which are dependent of many parameters difficult to calibrate. Without such a simplification, calibration would have been required because tendon compliance was found substantially higher in TD children and children with CP compared to the default values of the musculoskeletal models^23^. Calibration would also have been required if more than 3 biarticular muscles were modelled. *Gas*, *Se*, *Rf* are assimilated to their biarticular agonists group. We hypothesized that during passive mobilizations or gait, lengths and moment arms of the adjacent biarticular muscles follow the same evolution. We also hypothesized that, in the flexed positions, the biarticular muscles were slacked enough not to produce any resistance. We verified this assumption with a generic musculoskeletal model, for which biarticular muscles produce very few passive moments at the limits of the ROM^16^. In addition, the viscoelastic properties of the passive structures were neglected in our study^6,24^. The main reason for this methodological choice is that in children with CP, the velocity-dependency of the passive tension is experimentally complicated to study without triggering a reflex due to spasticity. This has likely led to a slight under-estimation of the passive moment and power during gait, in particular during swing phase where velocity is important. The proposed procedure and model may be also not adapted to study other activities with higher joint velocities, like running.

Knowingly, all choices and hypotheses were made in order to limit the number of joint mobilizations in the procedure and the number of parameters in the model.

### Passive contributions

In accordance with the literature about TD adults^7–9^, our results confirm that passive structures can contribute substantially at different instants of the gait cycle with differences between uni- and biarticular structures.

In terms of power contribution and share between uni- and biarticular structures, this article present results that are very close to the Whittington et al.’s^9^ ones, which suggest the consistency of our model, although the limited number of positions tested. Passive stretch of the biarticular muscles, particularly the *Gas* and *Rf*, contributed to the inter-segmental moments and powers seen in normal gait.

Another aspect highlighted by Whittington et al.^9^ and that we also found here is the transfer of energy via the bi-articular muscles. Indeed, these muscles, particularly the *Gas* and *Rf*, play role in the transfer of energy from one joint to another. At the knee, the absorption of energy by the *Rf* occurs at the same time as its generation at the hip, suggesting that this muscle transfers energy from one joint to another. Similarly, *Gas*, being stretched during the knee extension and ankle dorsiflexion (20–40% of the gait cycle), shall store elastic energy, which will be released when the knee flexes and the ankle plantarflexes (40–60% of the gait cycle), in a stretch-recoil mechanism.

However, while Whittington et al.^9^ did not find a significant role of passive *Se* stretch in normal walking, our analysis reveal that these ones could contribute at the knee and at the hip both during late swing. This difference may be due to the method, model or population studied.

This study also highlights some passive actions not described in the literature. For instance, the *Rf* seems to participate non-negligibly to the knee inter-segmental moment observed during early swing. Indeed, at this instant, the mean inter-segmental moment was fully explained by the *Rf* resistance. Moreover, at the ankle, a non-negligible passive extension moment is also present during the swing phase. Since the inter-segmental moment is null, it emphasizes that the dorsiflexor muscles have to produce non-minor flexion moments, of ~ 0.05 Nm/kg, to counteract this passive resistance.

The estimation of passive contribution to inter-segmental dynamics in gait of children with CP has not been made since Tardieu et al.’s^12^. They studied only the ankle joint and calculated the passive moment contribution only at one specific instant, approximately 55% of the stance phase. They showed a large variability amount the subjects, with some presenting large passive moment contributions and others only minor ones. They hypothesized that small contribution values indicate the presence of excessive contractions of the triceps surae muscle during gait, while large value indicate that gait was perturbed mainly because of a non-neural factor, i.e. the passive stiffness. Indeed, such a study of the passive moment contribution can enlighten on the impact of passive stiffness during gait and open prospect of a truly personalised treatment of passive stiffness that may lead to gait disorders in children with CP.

### Differences between TD and CP children

Results in CP children are more variable than in TD. Their passive contribution to inter-segmental moments or powers were different, not because of higher passive moment but because of lower inter-segmental ones. It is interesting to see that their passive moment during gait are, in mean, not different from the TD ones. Several hypothesis may explain those findings, either CP and TD do have the same maximum tolerance to passive tension or they do not have the capacity to produce more active moment with the antagonist muscles in order to overcome an increased passive moment that could result from the greater passive stretch required if they adopted the same kinematics as TD children^25^. Conversely, increased passive moment could also compensate for the weakness of the agonist active structures affected by different pathologies. This phenomenon was reported by Gaudreault et al.^26^, Lamontagne et al.^27^ and Siegler et al.^28^.

Two specific differences with TD children were found. The first difference concerns the impact of the *Gas* during late swing. Indeed, *Gas* seems not sufficiently stretched to produce passive moments because of the excessive plantarflexion (not resulting here from muscle retraction). Thus, the knee passive moment and power during late swing mainly come from the *Se*, whereas in TD children the contribution is shared between *Se* and *Gas*. The second difference is a negative passive power burst during loading response at the ankle, which is not present during TD gait. This passive power is due to the fore-foot initial contact corresponding to an initial stretch of the plantarflexor muscles and is mainly due to uniarticular structures of the ankle.

### Limitations

Besides limitations on the simplified musculoskeletal model discussed before (Section “Procedure and model”), other assumptions are made in this study. First, following the most common assumption in the literature, we assume that passive properties are additive with the active components present^8^. Second, the passive contribution to inter-segmental moments or powers values reported here were directly taken on the mean curves of the both populations in order to be able to compare our results with the literature. This should not mask the fact that variability is present among the subjects and particularly in children with CP. This variability is even a strong argument to evaluate subject-specifically the impact of passive stiffness on gait in children with CP and to better detail the pathology. Third, we have only considered passive solicitations during a flexion–extension movement. In the case of TD gait, this may be sufficient, since movements in other planes are minimal at the various joints. However, in the case of pathological gait, it might be interesting to consider the other planes, for which non-negligible moments can be determined. The use of a 3D passive moment model at the different joints, and especially at the hip, could prove to be beneficial although difficult to measure experimentally.

## Conclusion

In this study, we propose a procedure, requiring a minimal amount of passive stretching tests, and a model for the identification of the contribution to inter-segmental dynamics of passive mechanisms due to uni- and biarticular structures during gait. This method could therefore enable a longitudinal monitoring of the evolution of passive stiffness and its impact on gait. We have shown that passive mechanism contribute substantially at different instants of the gait cycle, mainly during the push-off and swing phases for the hip and the knee and the push-off for the ankle. In the case of CP children, we have found passive moments and power during gait relatively of the same amount as the TD ones but with a larger inter-subject variability and reduced inter-segmental moments. These results emphasize on the need to have a method that individualize the quantification of the passive stiffness. Understanding how and to what extent the musculo-articular complex responds passively to a solicitation can greatly improve our understanding of the mechanisms related to locomotion and better treat certain gait disorders. The proposed procedure and model enable a comprehensive assessment of the passive mechanisms with the aim to define a subject-specific treatment of the passive stiffness which contributes to impaired gait.

## Supplementary Information

Supplementary Information 1.

Supplementary Information 2.

Supplementary Information 3.

## Data Availability

The raw data used in this study are not publicly available due to national human research legislation. Data generated or analyzed during this study are available in Supplementary Material.

## References

[1] Amankwah K, Triolo RJ, Kirsch R (2004). Effects of spinal cord injury on lower-limb passive joint moments revealed through a nonlinear viscoelastic model. JRRD.

[2] Edrich T, Riener R, Quintern J (2000). Analysis of passive elastic joint moment in paraplegics. IEEE Trans. Biomed. Eng..

[3] Mansour JM, Audu ML (1986). The passive elastic moment at the knee and its influence on human gait. J. Biomech..

[4] Riemann BL, DeMont RG, Ryu K, Lephart SM (2001). The effects of sex, joint angle, and the gastrocnemius muscle on passive ankle joint complex stiffness. J Athl. Train.

[5] Riener R, Edrich T (1999). Identification of passive elastic joint moments in the lower extremities. J. Biomech..

[6] Nordez A (2006). Caractérisation et Modélisation du Comportement Mécanique du Complexe Musculo-Articulaire en Conditions Passives.

[7] Gasparutto X, Jacquelin E, Dumas R (2018). Contribution of passive actions to the lower limb joint moments and powers during gait: A comparison of models. Proc. Inst. Mech. Eng..

[8] Koussou A, Desailly E, Dumas R (2021). Contribution of passive moments to inter-segmental moments during gait: A systematic review. J. Biomech..

[9] Whittington B, Silder A, Heiderscheit B, Thelen DG (2008). The contribution of passive-elastic mechanisms to lower extremity joint kinetics during human walking. Gait Posture.

[10] Desloovere K (2006). Do dynamic and static clinical measurements correlate with gait analysis parameters in children with cerebral palsy?. Gait Posture.

[11] Papageorgiou E (2019). Are spasticity, weakness, selectivity, and passive range of motion related to gait deviations in children with spastic cerebral palsy? A statistical parametric mapping study. PLoS ONE.

[12] Tardieu C, Lespargot A, Tabary C, Bret MD (1989). Toe-walking in children with cerebral palsy: Contributions of contracture and excessive contraction of triceps surae muscle. Phys. Ther..

[13] Wu G (2002). ISB recommendation on definitions of joint coordinate system of various joints for the reporting of human joint motion—part I: Ankle, hip, and spine. J. Biomech..

[14] Desailly E (2008). Analyse Biomécanique 3D de la Marche de l’enfant Déficient Moteur: Modélisation Segmentaire et Modélisation Musculo-squelettique.

[15] Doriot N, Cheze L (2004). A three-dimensional kinematic and dynamic study of the lower limb during the stance phase of gait using an homogeneous matrix approach. IEEE Trans. Biomed. Eng..

[16] Lai AKM, Arnold AS, Wakeling JM (2017). Why are antagonist muscles co-activated in my simulation? A musculoskeletal model for analysing human locomotor tasks. Ann. Biomed. Eng..

[17] Modenese L, Barzan M, Carty CP (2021). Dependency of lower limb joint reaction forces on femoral version. Gait Posture.

[18] Kainz H, Schwartz MH (2021). The importance of a consistent workflow to estimate muscle-tendon lengths based on joint angles from the conventional gait model. Gait Posture.

[19] Diong JHL (2012). Passive mechanical properties of the gastrocnemius after spinal cord injury. Muscle Nerve.

[20] de Vlugt E (2010). The relation between neuromechanical parameters and Ashworth score in stroke patients. J. NeuroEngineering Rehabil..

[21] Derrick TR (2020). ISB recommendations on the reporting of intersegmental forces and moments during human motion analysis. J. Biomech..

[22] Michaud F, Shourijeh MS, Fregly BJ, Cuadrado J (2020). Do muscle synergies improve optimization prediction of muscle activations during gait?. Front. Comput. Neurosci..

[23] Veerkamp K (2022). Personalisation of plantarflexor musculotendon model parameters in children with cerebral palsy. Ann. Biomed. Eng..

[24] Singer BJ, Dunne JW, Singer KP, Allison GT (2003). Velocity dependent passive plantarflexor resistive torque in patients with acquired brain injury. Clin. Biomech..

[25] Koussou A, Dumas R, Desailly E (2022). Can passive stiffness explain kinematics disorders in children with cerebral palsy?. Gait Posture.

[26] Gaudreault N, Gravel D, Nadeau S (2009). Evaluation of plantar flexion contracture contribution during the gait of children with Duchenne muscular dystrophy. J. Electromyogr. Kinesiol..

[27] Lamontagne A, Malouin F, Richards CL (2000). Contribution of passive stiffness to ankle plantarflexor moment during gait after stroke. Arch. Phys. Med. Rehabil..

[28] Siegler S, Moskowitz GD, Freedman W (1984). Passive and active components of the internal moment developed about the ankle joint during human ambulation. J. Biomech..

