# Supplementary material for: A procedure and model for the identification of uni- and biarticular structures passive contribution to inter-segmental dynamics
Source: Sci Rep. 2023 Jun 29;13:10535. doi: 10.1038/s41598-023-37357-w (PMC10310719; doi:10.1038/s41598-023-37357-w)

**Supplementary material**

S1 - Exponential models:

The relationship between passive moments and sagittal joint angles, θ, was modelled with double exponentials:

$$Passive Moment= C_{1}+C_{3}+C_{0}$$

$$\left\{ \begin{aligned} C_{1}= \frac{E_{1}}{\alpha_{1}}\left( e^{\alpha_{1}\left( \theta-\theta_{1} \right)}-1 \right) if \theta>\theta_{1} \\ C_{1}= 0 if \theta\leq\theta_{1} \\ \begin{matrix} C_{3}= \frac{E_{3}}{\alpha_{3}}\left( e^{\alpha_{3}\left( \theta_{3}-\theta\right)}-1 \right) if \theta<\theta_{3} \\ C_{3}= 0 if \theta\geq\theta_{3} \end{matrix} \end{aligned} \right.$$

where $\alpha_{1,3}$ represent index of passive stiffness (slope of the linear relationship between stiffness and passive moment), $E_{1,3}$ correspond to the stiffness for zero moment (intercept of the linear relationship between stiffness and passive moment) and $\theta_{1,3}$ are the angles at which the joint develops a passive moment in one direction. $C_{0}$ is an offset passive moment term serving purely as a shaping parameter for the stiffness model.

The relationship between passive forces and MTU lengths, $l$, was modelled with a simple exponential:

$$\left\{ \begin{aligned} Passive Force= \frac{E_{f}}{\alpha_{f}}\left( e^{\alpha_{f}\left( l-l_{0} \right)}-1 \right) if l>l_{0} \\ Passive Force=0 if l\leq l_{0} \end{aligned} \right.$$

where $\alpha_{f}$and $E_{f}$ are parameters that concerns MTU stiffness, and $l_{0}$ is the MTU slack length.

S2 - Quality of the modelling:

Quality of the modelling was evaluated with average root mean square errors (RMSE) and coefficient of determination (R²) between experimental and modelled data. For TD children, average RMSE were 0.009±0.002 Nm/kg for ${MA}_{Uni}$, 0.282±0.112 N/kg for $F_{Gas}$, 0.015±0.008 Nm/kg for ${MK}_{P3'}$, 0.010±0.004 Nm/kg for ${MK}_{P4'}$, 0.368±0.154 N/kg for $F_{Se}$, 0.367±0.257 N/kg for $F_{Rf}$ and 0.031±0.023 Nm/kg for ${MH}_{Uni}$. Average R² were 0.95±0.02 for ${MA}_{Uni}$, 0.90±0.03 for $F_{Gas}$, 0.91±0.05 for ${MK}_{3'}$, 0.96±0.03 for ${MK}_{P4'}$, 0.93±0.04 for $F_{Se}$, 0.94±0.07 for $F_{Rf}$ and 0.93±0.06 for ${MH}_{Uni}$

For CP children, average RMSE were 0.009±0.003 Nm/kg for ${MA}_{Uni}$, 0.241±0.103 N/kg for $F_{Gas}$, 0.017±0.007 Nm/kg for ${MK}_{P3'}$, 0.012±0.005 Nm/kg for ${MK}_{P4'}$ 0.473±0.219 N/kg for $F_{Se}$, 0.432±0.180 N/kg for $F_{Rf}$ and 0.034±0.017 Nm/kg for ${MH}_{Uni}$. Average R² were 0.91±0.05 for ${MA}_{Uni}$ , 0.89±0.04 for $F_{Gas}$, 0.94±0.04 for $M_{P3'}$, 0.95±0.02 for ${MK}_{P4'}$ 0.93±0.04 for $F_{Se}$, 0.94±0.03 for $F_{Rf}$ and 0.92±0.03 for ${MH}_{Uni}$.

Examples for a typical TD child and a child with PC are given in Figures S2-1 and S2-2. Individual quality of the modelling can be found in the Supplementary Material 2. Low RMSE and high R² values are found for the different subjects, indicating a good quality of the modelling.

Figure S2-1: Experimental and modelled by an exponential of the passive moment model in a typical TD child. Root mean square error (RMSE) is also express to evaluate the quality of the exponential model.


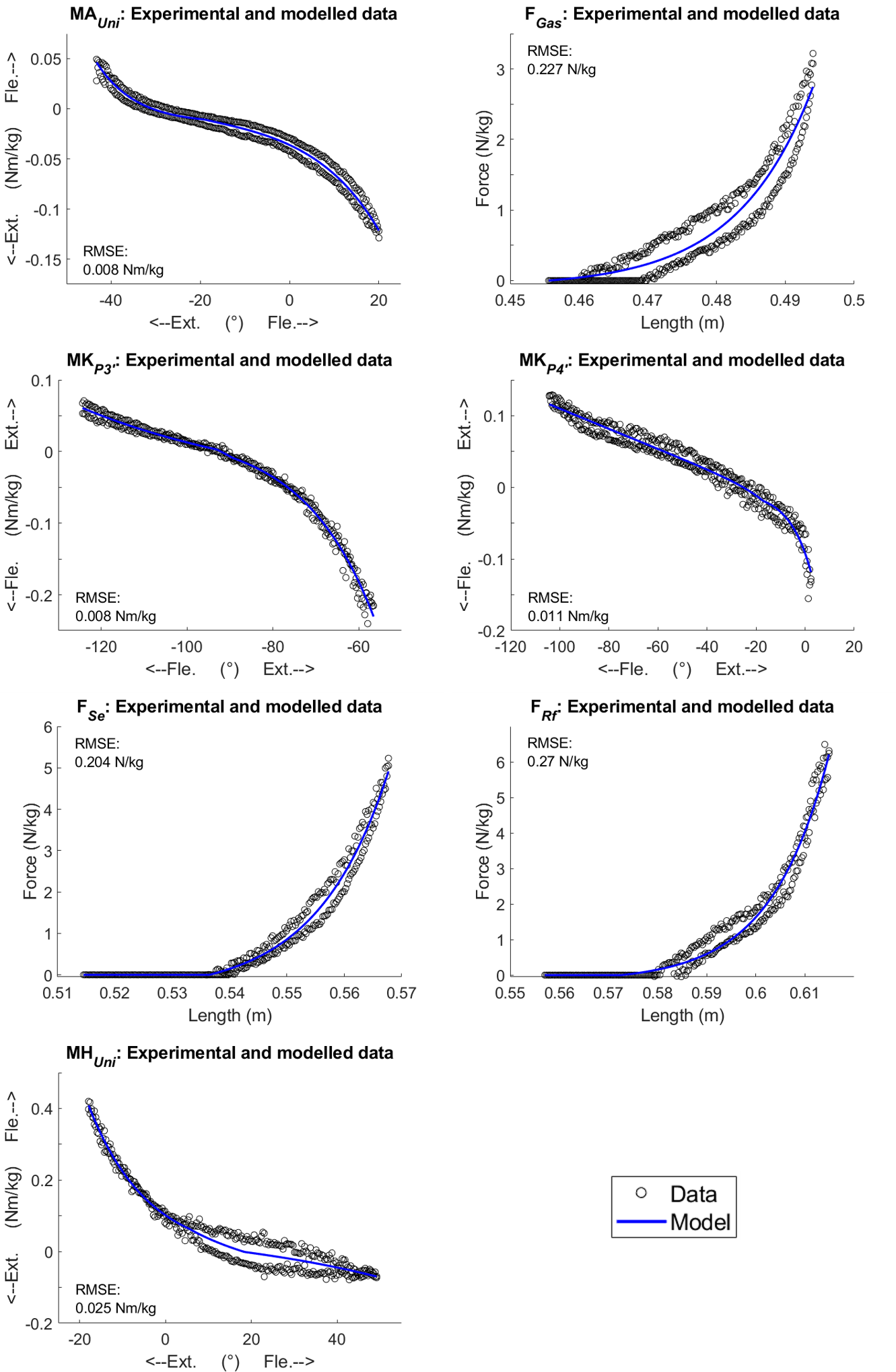


Figure S2-2: Experimental and modelled by an exponential of the passive moment model in a typical CP child. Root mean square error (RMSE) is also express to evaluate the quality of the exponential model.


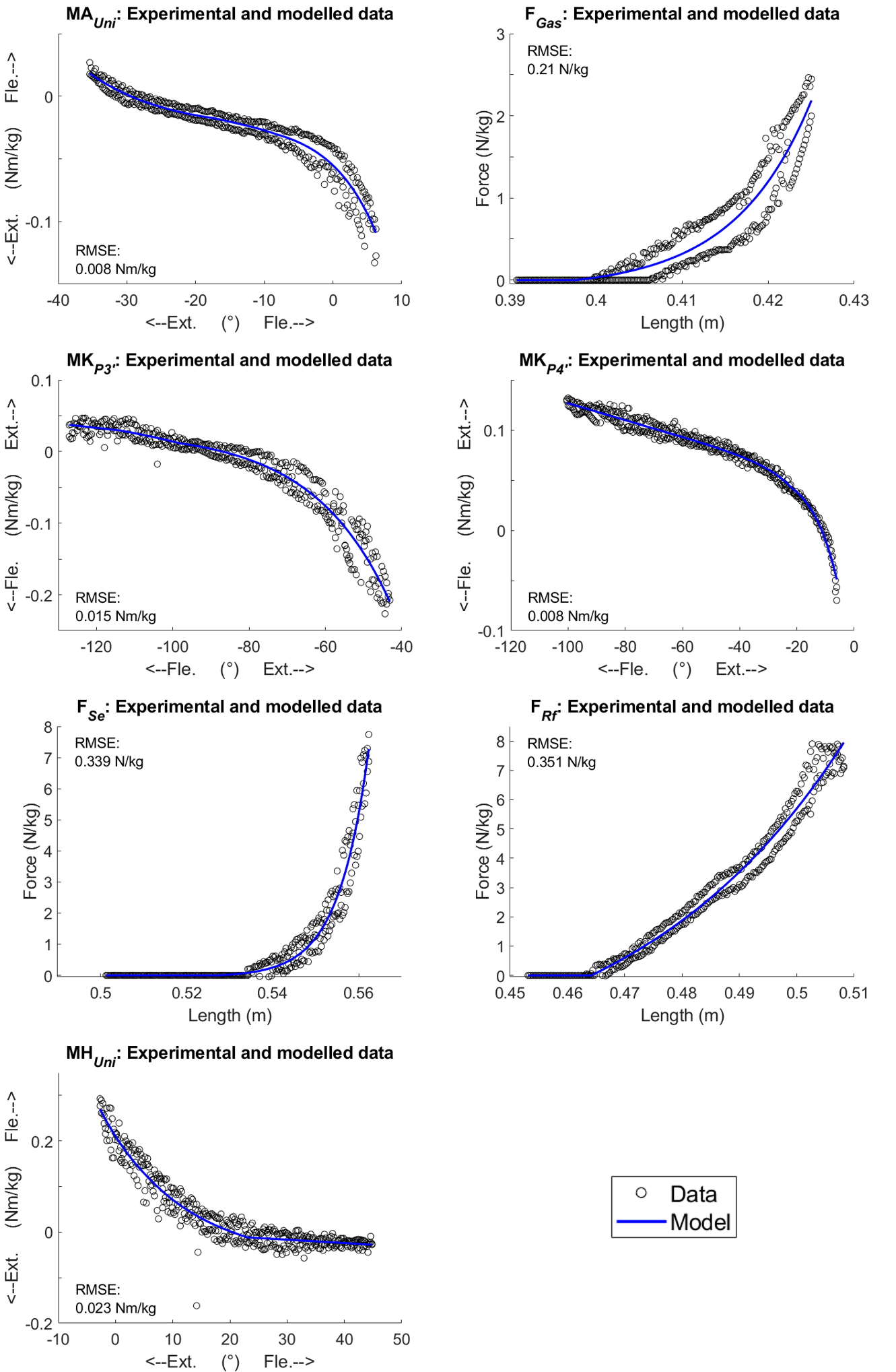

Supplement: Supplementary file 1 — Supplementary Information 1. [file 41598_2023_37357_MOESM1_ESM.docx]
